# Simulation and experimental research of electric tractor drive system based on Modelica

**DOI:** 10.1371/journal.pone.0276231

**Published:** 2022-11-17

**Authors:** Yawei Mao, Yiwei Wu, Xianghai Yan, Mengnan Liu, Liyou Xu

**Affiliations:** 1 College of Vehicle and Traffic Engineering, Henan University of Science and Technology, Luoyang, Henan, China; 2 State Key Laboratory of Power System of Tractor, YTO Group Corporation, Luoyang, Henan, China; Beijing Institute of Technology, CHINA

## Abstract

The electric tractor has the advantages of zero-emission, high efficiency, and low noise, which is the direction of future development and transformation of agricultural power machinery. Aim at the problem that the simulation methods commonly used in the development of electric tractor drive system are poorly generalized and cannot meet the simulation needs of complex multi-domain physical systems. This paper proposes a modeling method for an electric tractor drive system, takes the YTO-500 tractor as the research object, designs and calculates the overall scheme and parameters of its drive system, divides the drive system into modules, establishes the energy system, motor system and mechanical parts model based on Modelica, and integrates the simulation model of electric tractor drive system on this basis. The traction performance and transportation working conditions were simulated and tested. With compared and analyzed, in the traction characteristics, the simulation and test results of maximum speed, maximum traction force, and maximum traction power of each gear are consistent; within 400s transportation simulation conditions, the speed range of electric tractor is 13~28km·h^-1^, which is consistent with the speed range of electric tractor transportation gear. The results show that the simulation and the test results are consistent, which verifies the credibility of the simulation and the correctness of the model built, providing a basis for future research and development of agricultural machinery.

## Introduction

With the deterioration of the environment and the intensification of energy depletion, it is forward-looking and necessary to develop environmentally friendly, resource-saving, and efficient agriculture. Compared to traditional tractors, electric tractors offer higher efficiency, lower air pollution, and can reduce noise pollution. Various countries around the world proposed agricultural machinery production to focus on developing resource-saving, environmentally friendly, and mechanized, improving the state of the technology of agricultural machinery, improving the efficiency of energy use, and promoting energy conservation and emission reduction [1–4].

Throughout the development history of electric tractors for more than 100 years, the research on electric tractors can be divided into three stages. In the early stages, use of the power grid to provide energy. For example, the Siemens company in Germany produced the earliest electric tractors for simple drive rototiller operations [5], the former Soviet Union created the first tracked electric tractor [6], and Switzerland’s Grunder develops walk-behind electric tractor for multiple operations [7]. In the middle stage, with the development of battery technology and power electronic control technology, electric tractors by the on-board battery system provide energy. For example, General Electric of the United States has released the Elec-Trak series of electric tractors driven by lead-acid batteries with permanent magnet DC brushless motors [8], Electric Tractor, a Canadian company, produced the Electric Ox series of electric tractors consisting of six lead-acid batteries as an energy system, with separate electric motors for the drive wheels and implements [9]. At the current stage, with the development of intelligent algorithms and automation technology, the design and application of electric tractors have the trend of intellectual development, promoting the development of new products of electric tractors, and providing help for the development of intelligent agricultural equipment products of new energy.

At present, the related technology of electric tractors is one of the hot issues researched by scholars at home and abroad in recent years. Foreign research on electric tractors has been more important. Yuko UEKA [4] converted an internal combustion engine tractor into an electric tractor with an AD motor and studied the required energy consumption. Arjharn [10, 11] modified an electric tractor based on a diesel tractor and studied the energy consumption and traction characteristics of this electric tractor. Seung-Yun Baek [12] designed and developed an electric four-wheel drive tractor and analyzed its agricultural and traction performance. Rodnei Regis de Melo [13] proposed a solution for automatic slip control of a two-wheel drive electric tractor to improve the traction efficiency of the electric tractor. The domestic research on electric tractors has just started. Huisong Gao [14, 15] built a simulation platform for electric tractors by secondary development of Advisor top-level module. Mengnan Liu [16] and Liyou Xu [17] established a simulation platform for an extended range electric tractor based on CRUISE. Xin Zhang [18] used Simulink to establish a pure electric tractor transmission system for simulation research. Nowadays, computer technology for simulation analysis has become an essential tool. Qiang Sun [19] proposed a simulation method based on CRUISE/Simulink for the dozing conditions of the crawler dozer, and Zhuowei Chen [20] designed and verified the control algorithm based on the joint simulation of Carsim and Simulink, both of which obtained good simulation results. Among them, MATLAB/Simulink is commonly used for off-road vehicle simulation [21–24] and can be used to design and verify the robustness and real-time performance of the algorithms [25–27]. However, AVL CRUISE, Advisor, CarSim, MATLAB/Simulink, and other performance simulation software focus on the modeling and simulation of a single component or a single system, which is hard to achieve unified modeling and simulation in multiple domains. Therefore, when modeling and simulating complex systems in multiple domains, most of them take the approach of multiple software modeling and then joint simulation, failing to complete the overall system modeling and simulation under a unified modeling environment, which will lead to a large deviation between simulation results and actual results. In addition, they are more suitable for the development and performance analysis of road vehicles like passenger cars and commercial vehicles [28–31]. At the same time, MATLAB/Simulink is limited by modeling mechanisms such as graphical representation, causal derivation, and algebraic loop decomposition, resulting in a very tedious and complex modeling process. The Modelica language was proposed in this background as an object-oriented, non-causal, multi-domain unified modeling language to solve complex physical system modeling and simulation problems [32–34]. The Modelica language describes the physical processes of each subsystem in different domains based on mathematical equations, realizes model composition and integration according to the topology of the physical system based on the principle of intrinsic component connectivity of the language, and realizes the simulation operation of the integrated system by solving the differential algebraic equations of the system [35, 36]. Modelica, a multi-domain modeling language, uses a non-causal, textual formulation and image-compatible modeling and simulation mechanism, which is well suited to avoid deviations of simulation results from experimental measurements. In addition, when modeling based on the Modelica language, the system is divided into subsystems of different domains, which can then be written by professionals with knowledge of that domain, thus increasing the accuracy of the modeling. The Modelica language has the characteristics of parametric, modular, and graphical, which makes the system modularity both independently buildable and allows the rapid assembly of components from different domain model libraries, for tractor transmission systems can develop a scalable, cause-free, flexible, and reusable model library to meet various research purposes. It contributes to prototype implementation, improves system modeling capabilities, and reduces the time to build and verify models. OpenModelica is an open source simulation platform based on the Modelica language, which establishes a library of models based on the Modelica language containing mechanical, electronic, and control domains, providing the basis for the realization of modeling and simulation of electric tractor drive systems [37].

This paper takes the YTO-500 tractor as the research object, establishes the simulation model of the electric tractor drive system based on the Modelica standard library, VehicleInterface library, and OpenModelica simulation platform, and verifies the credibility of the simulation and the correctness of the model by comparing and analyzing the performance of the drive system simulation and the test results of the vehicle.

### Overall scheme of electric drive system

**Fig 1:**
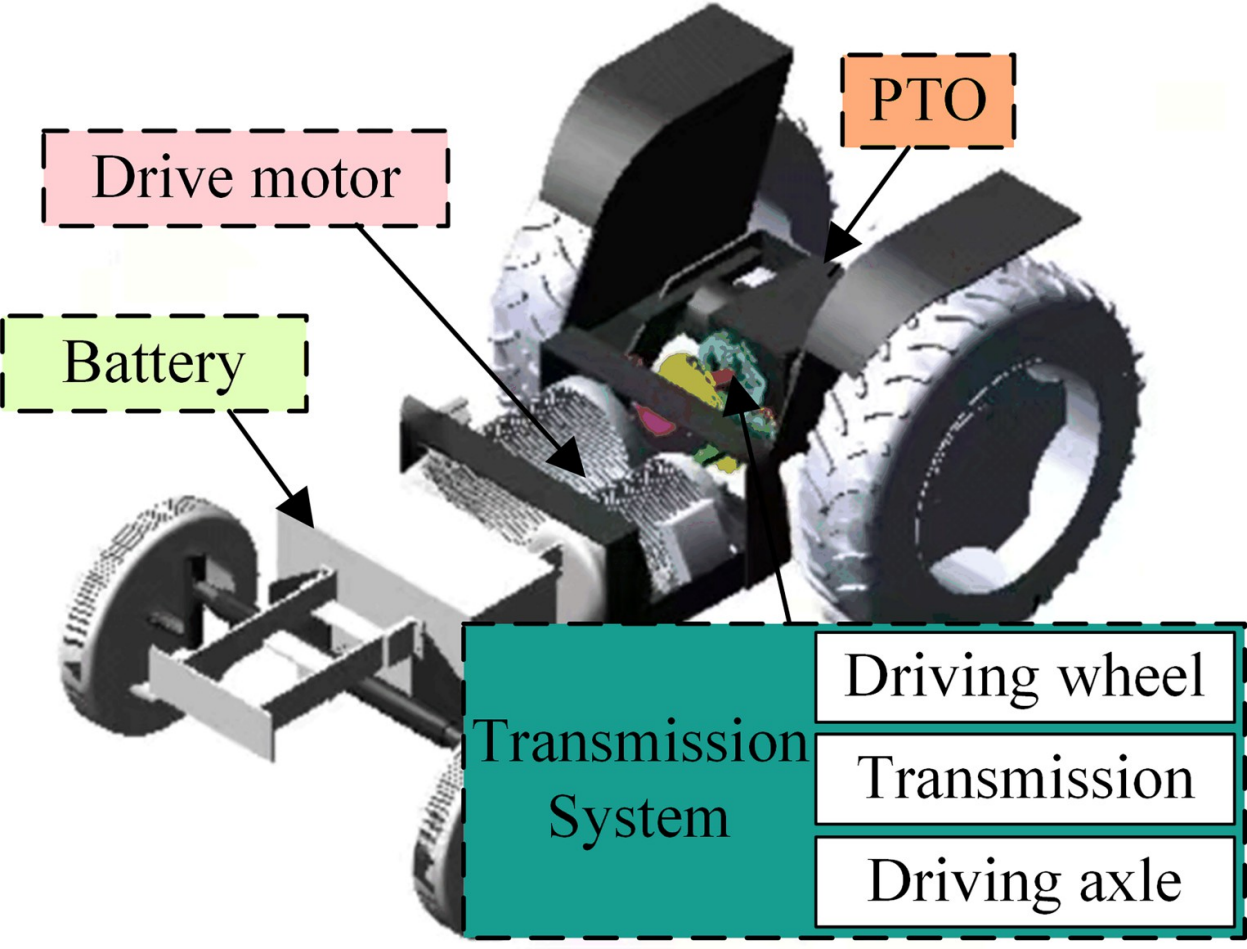
Powertrain diagram of electric tractor.

According to the different operating environments and working objects, there are various structural solutions for the drive system of electric tractors. Considering the usage and operating environment of the electric tractor, if it adopts a mass of electronic components and electronic control system, its reliability and safety are difficult to guarantee [38]. Combining the development of domestic electric vehicles using electric motors and the current situation of domestic research on electric tractors, this paper designs the electric drive system for the YTO-500 tractor, and Fig 1 shows the powertrain diagram of electric tractor.

The main components of the electric tractor powertrain in Fig 1 include the battery pack, drive motor, transmission system, and power take off (PTO), where the transmission system consists of the transmission, drive axle, and drive wheels. The transmission input shaft has a clutch to turn on and turn off the power from the motor to the wheels. The transmission output power transfers to the drive wheels via the drive axle. The motor adopts DC motor, which has excellent electromagnetic torque control characteristics [39], good speed regulation performance, and large starting torque, which can meet the needs of electric tractor traction operation [40]. The battery adopts a lead-acid battery suitable for electric tractors due to its low price and high production volume [41]. This form of the electric tractor drive system is less improved than the traditional tractor drive system, is convenient for the drive system transformation, and can be used as a vehicle for electric tractor performance analysis, contributing to promoting the marketization of electric tractors.

### Parameter design of electric drive system

When the drive system is specifically designed, first of all, from the aspect of deciding each performance demand of electric tractor, according to its mission statement in the performance index requirements, in the case of meeting the functional demand of electric tractor, design the corresponding key parameters of rated tractive force, drive motor, energy system, and transmission system.

### Rated pull

When the electric tractor traction operation, by the traction resistance and rolling resistance together, and the traction resistance for the main resistance, to overcome the traction resistance to indicate the rated traction force.

Rated traction force is used as design input when higher traction performance is required. The conditions of maximum traction force provided by the rated traction force are that the tractor has suitable stubble land in the horizontal section, the tractor with basic plowing speed, and the drive wheel slip rate is equal to the characteristic slip rate.

Plowing operation is the most commonly used tractor is also the most heavily loaded operation, so the tractor’s rated pull is determined by the average drag force of the plowing operation, that is:

$$F_{t}=zb_{1}h_{k}k$$ (1)

In the formula: *F*_*t*_、*Z*、*b*_1_、*h*_*k*_、*k* are the average drag force, the number of plowshares, the width of a single plowshare, the tillage depth, and soil specific resistance, respectively.

The tractor’s operating environment is complex and changeable. Agricultural equipment by resistance will produce certain changes. Consider the tractor in the operating process of these factors’ changes will cause the instability of traction resistance, should retain 10%~20% of the reserve traction, that is:

$$F_{tN}=(1.1∼1.2)F_{t}$$ (2)

In the formula: *F*_*tN*_ is the rated drag force of the tractor, *F*_*t*_ is the average drag force.

### Driving motor

Due to the specificity of the operating environment, the electric tractor drive motor requires the following characteristics:

1. The drive motor has large starting torque and strong overload capacity to meet sudden changes in working load.
2. A wide speed range can adapt to slow plowing operations and fast transport.
3. The drive motor has strong dust-proof and waterproof capability to adapt to the complex and harsh operating environment.

The traction force required by the electric tractor comes from the drive motor. As the drive motor has low-speed constant torque and high-speed constant power characteristics and the electric tractor speed and traction requirements, the power of the drive motor should meet:

$$P_{TN}\geq \frac{F_{t}V_{T}}{3600\eta_{\tau }}$$ (3)

In the formula: *P*_*TN*_ is the rated power of the drive motor, *F*_*t*_ is the average drag force, *V*_*T*_ is the speed of the tractor plowing operation, *η*_*τ*_ is the traction efficiency.

The traction efficiency of electric tractors is related to the transmission efficiency of the drive system, the slip efficiency and rolling efficiency of the drive wheels, and the efficiency of the drive motor and controller, that is:

$$\eta_{\tau }=\eta_{mc}\eta_{m}\eta_{\delta }\eta_{f}$$ (4)

In the formula: *η*_*τ*_ is the traction efficiency of the electric tractor, *η*_*mc*_ is the transmission efficiency of the drive system, *ηδ* is the slip efficiency of the drive wheel, *η*_*f*_ is the rolling efficiency, *η*_*m*_ is the efficiency of the drive motor and controller.

According to the rated power, determine the rated speed and rated torque of the drive motor in the product catalog.

### Energy system

Power battery as electric tractor energy storage and energy supply device provides operating power for electric tractor, which needs to generate additional power for the vehicle load, electronic systems, and auxiliary equipment. The determination of the number of its battery pack should follow two principles: battery output power is greater than the maximum power of the drive motor; battery energy to meet the requirements of continuous operation time. Therefore, the rated power and the rated capacity of the energy system determine the number of cells in the power pack.

### Rated power

To meet the maximum power of the drive motor, the output power of the battery as an energy supply device needs to be greater than the maximum power of the drive motor.


$$N_{1}\geq \frac{P_{TM}}{P_{b}\eta_{mc}}$$ (5)

In the formula: *N*_1_ is the number of batteries, *P*_*TM*_ is the maximum power of the drive motor, *P*_*b*_ is the maximum output power of a single battery, *η*_*mc*_ is the efficiency of the drive motor controller.

### Rated capacity

The power battery provides all the energy of the electric tractor, so the power battery should meet the total energy demand of the electric tractor. The designed duration of this electric tractor determines the total energy of the power battery, which means the total battery energy is greater than the energy consumed by the rated operating time.


$$N_{2}\geq \frac{(P_{TN}+P_{l})T_{N}}{P_{b}}$$ (6)

In the formula: *N*_2_ is the number of batteries, *P*_*TN*_ is the rated power of the drive motor, *P*_*l*_ is the power of the accessory electrical components, *P*_*b*_ is the maximum output power of a single battery, *T*_*N*_ is the rated working time, which is 4 hours.

The number of batteries in the energy system, taking the larger value between *N*_1_ and *N*_2_, is *N* = max(*N*_1_,*N*_2_).

### Transmission system

Electric tractors are used for a wide range of applications and have different requirements for driving speed and drive torque. The speed required for various operations and the operating speed for different plowing is low, while the transport operation requires a faster-operating speed. Therefore, the calculation of the transmission ratio can be based on the rated torque and rated speed of the drive motor to determine the total transmission ratio of the drive system.


$$i_{dI}=\frac{F_{tN}r_{q}}{T_{mT}\eta_{d}}$$ (7)

$$i_{d\mathrm{II}}=0.377\frac{\omega_{mT}r_{q}}{v_{t}}$$ (8)

In the formula: *i*_*d*I_ is the total transmission ratio of the transmission system, *i*_*d*II_ is the speed ratio of the transmission gear, *F*_*tN*_ is the rated drag force of the electric tractor, *T*_*mT*_ is the rated torque of the drive motor, *η*_*d*_ is the transmission efficiency of the transmission element, *r*_*q*_ is the radius of the drive wheel, *ω*_*mT*_ is the rated speed of the drive motor, *v*_*t*_ is the driving speed of the electric tractor when it is working.

### Main parameters

Taking the YTO-500 tractor as the object, calculate the parameters of each component of the drive system according to the design of the above main parameters. The original parameters of the tractor using gravity of 25kN, the wheelbase is 1.975m, distance from the front counterweight centroid to the front axle of 0.083m, rated power is 36.8kW, rated torque is 159.7N·m, driven wheel radius is 0.375m, drive wheel radius is 0.63m. Equipped with a 1L-235 medium-sized plow, the single plow width is 3cm, and the plowing depth is 18cm~26cm. Table 1 shows the main parameters of the electric tractor calculated according to Eqs (1)–(8).

**Table 1:** Main parameters of electric tractor.

System	Parameter	Value
Whole system	Tractor gravity *W*_*t*_/N	18310
	Horizontal distance between centroid and driving wheel a/m	1.60
	Rated pull *F*_*tN*_/N	12000
Drive motor	Power rating *P*_*TN*_/kW	36.8
	Rated torque *T*_*Mt*_/N·m	216
	Rated speed *ω*_*Mt*_/r·min^-1^	1627
	Maximum speed *ω*_*max*_/r·min^-1^	2861
	Rated voltage *V*_*mT*_/v	380
Transmission System	Number of gears	4
	Gear ratio	1.3
	Overload transmission ratio	7.1
	Moderate load transmission ratio	5.4
	Light load transmission ratio	4.2
	Transmission ratio of transport gear	3.2
Energy system	Single rated capacity/Ah	100
	Single rated voltage/V	9.6
	Battery connection mode	3 Parallel connection 40 series connection
	Duration of plowing /h	3.75

### Electric drive system model establishment

**Fig 2:**
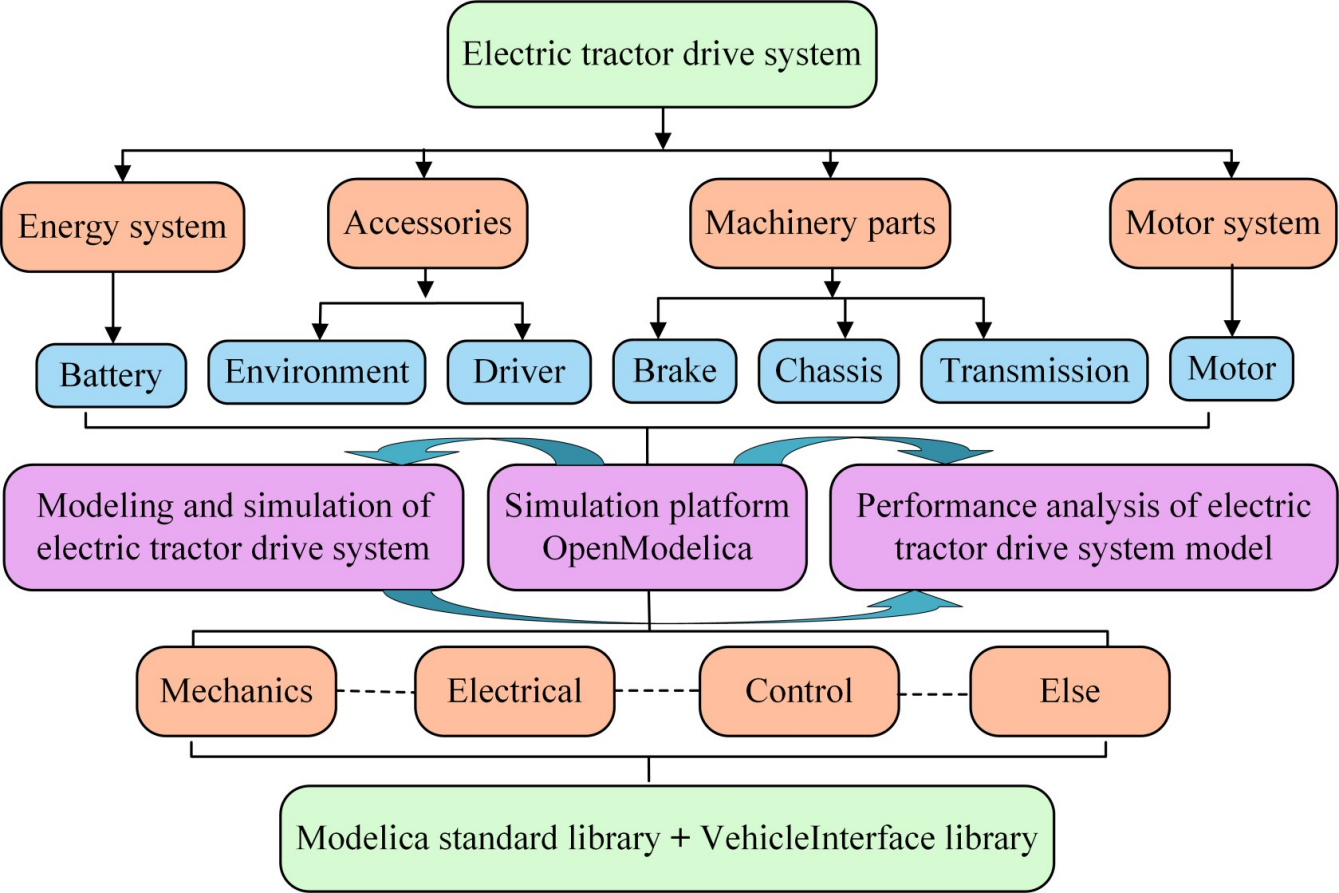
Module division of electric tractor drive system.

Based on the study of the basic principle of the electric tractor drive system and the existing modeling methods, according to the different subject areas to which the electric tractor drive system belongs, corresponding to the Modelica standard library in the field of machine, electricity, control and other sub-libraries, respectively, to its module division and based on the OpenModelica simulation platform to build subsystem models and integrate the simulation model of the electric tractor drive system. Fig 2 shows the module division of the electric tractor drive system.

In Fig 2, the electric tractor drive system decomposes into an energy system module, motor system module, mechanical component module, and accessory module. The energy system refers to the battery, the motor system module refers to the motor, and the mechanical components module mainly includes the transmission module, chassis module, brake module, and drive axle module. The accessory modules include driver, road, and environment.

### Subsystem models

The VehicleInterface library is developed based on the Modelica language and provides a basis for modeling. The model library allows the user to customize it with Modelica functions, and the model can be changed internally by the user entering the appropriate parameters. Since the VehicleInterface library contains both the battery model and the motor model, it only needs to be inherited and changed.

### Brake model

**Fig 3:**
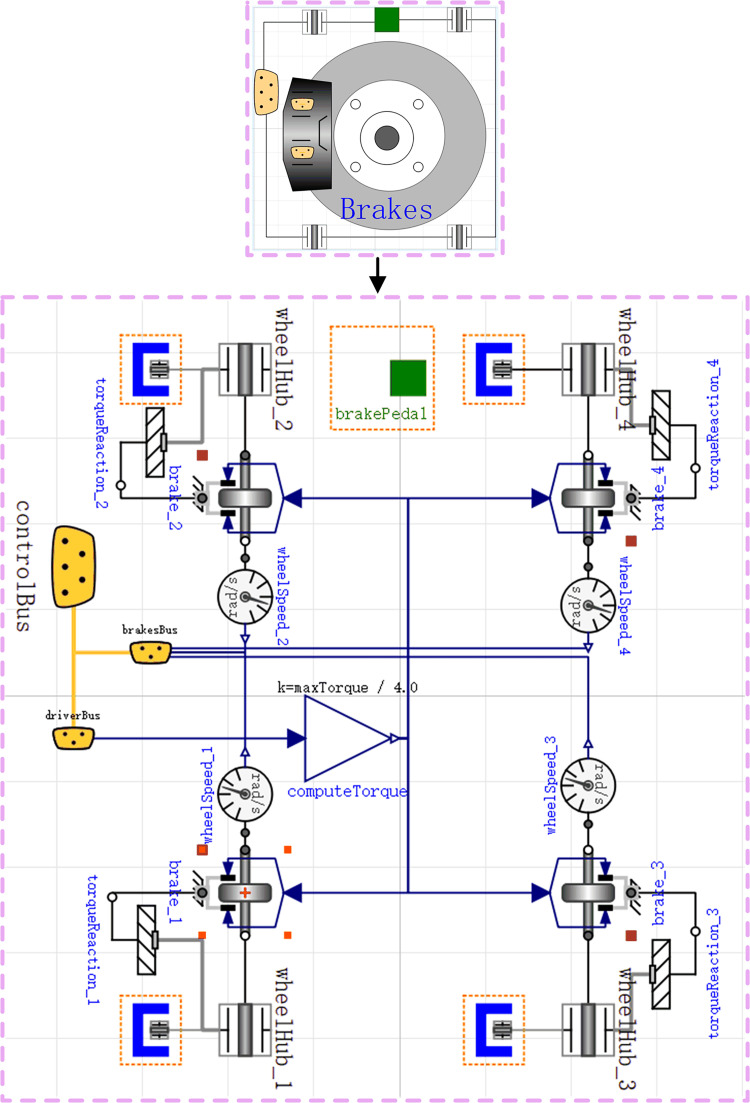
Brakes model.

Based on the physical structure of the brake and the braking principle, using the fundamental components Brake, FlangeWithBearing, ConstantTorque, and Mounting1D in the Modelica standard library to construct the brake model. Fig 3 shows the brakes model.

### Chassis model

**Fig 4:**
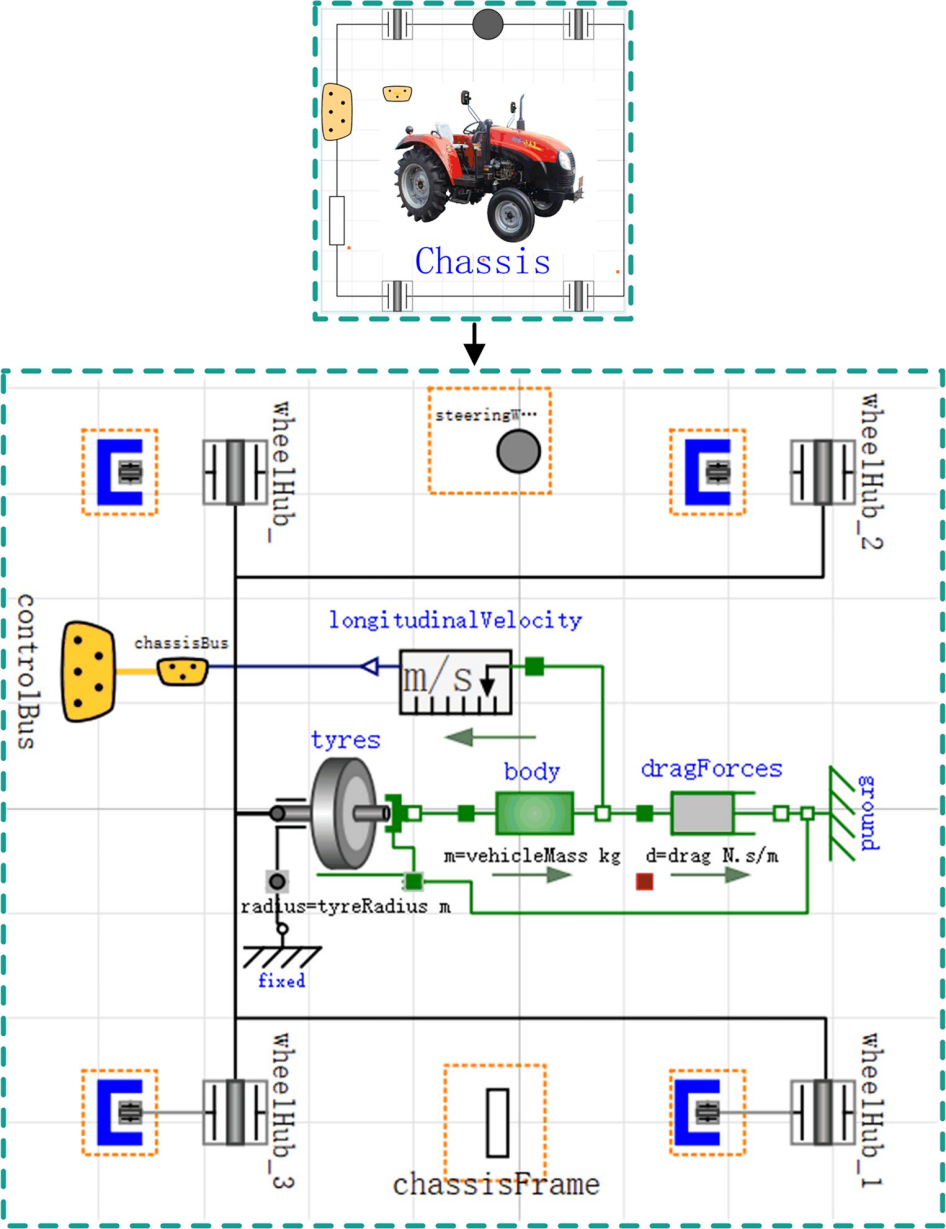
Chassis model.

As the foundation of the vehicle architecture, the chassis not only supports the on-board energy storage power supply, the electric motor, and the required auxiliary devices but also transmits and distributes the drive power. The chassis model consists of the drive wheels and the vehicle body model. When describing and modeling the chassis assembly using the Modelica language, the body is modeled as a single mass block and rigidly connected to the drive wheels. Fig 4 shows the chassis model.

### Transmission model

**Fig 5:**
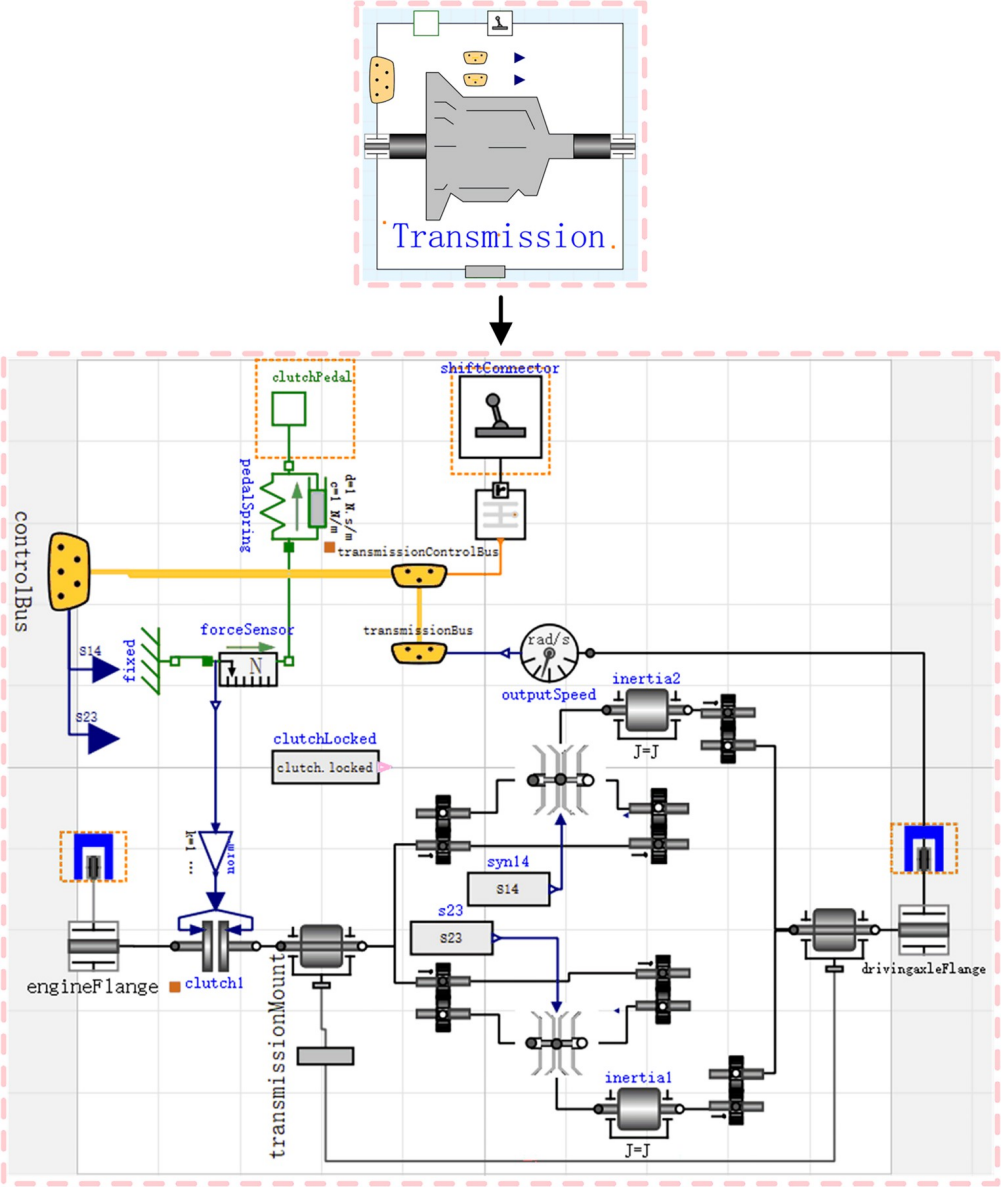
Transmission model.

**Fig 6:**
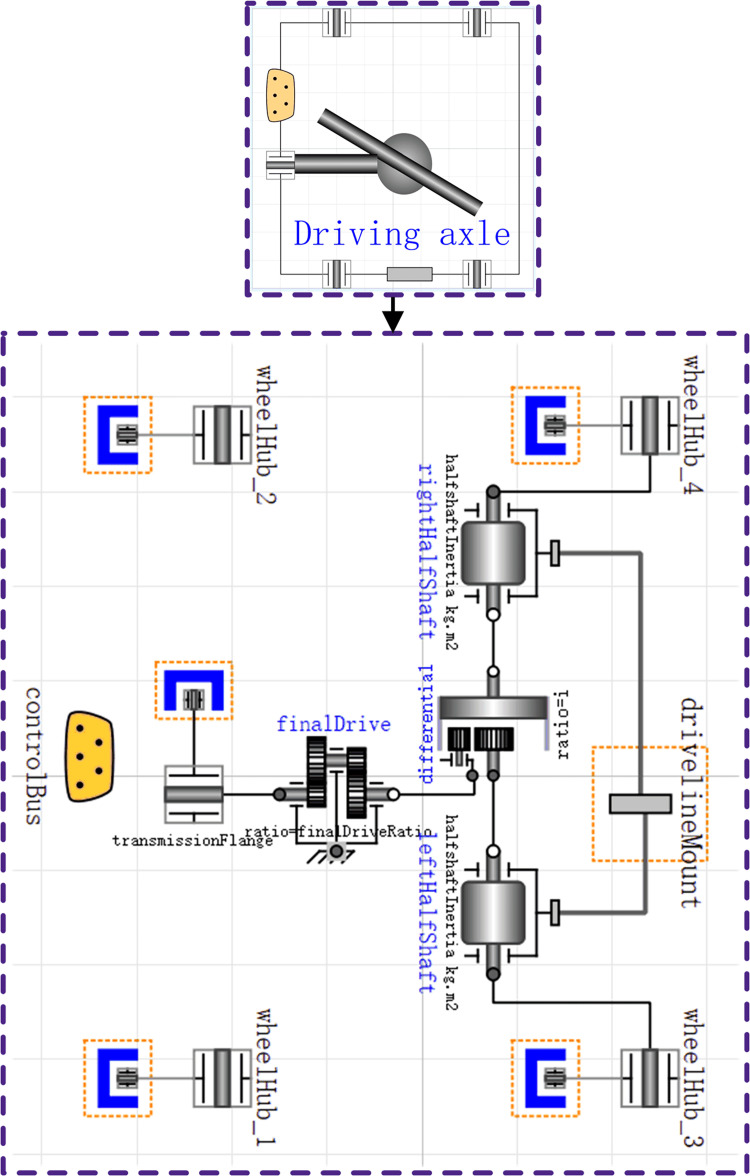
Driving axle model.

The gearbox and drive axle transmits the power in the form of mechanical energy to the rest of the vehicle. Since the rotational inertia of the rotating gear causes mechanical energy loss during operation, simulate the rotational inertia using the Inertia component of the Modelica library. When selecting the transmission ratio, it is required to control the operating state of the clutch. Fig 5 shows the built transmission model, where the mechanical energy output through the gearbox is input to the driving axle. Fig 6 shows the driving axle model.

### Electric drive system simulation model

**Fig 7:**
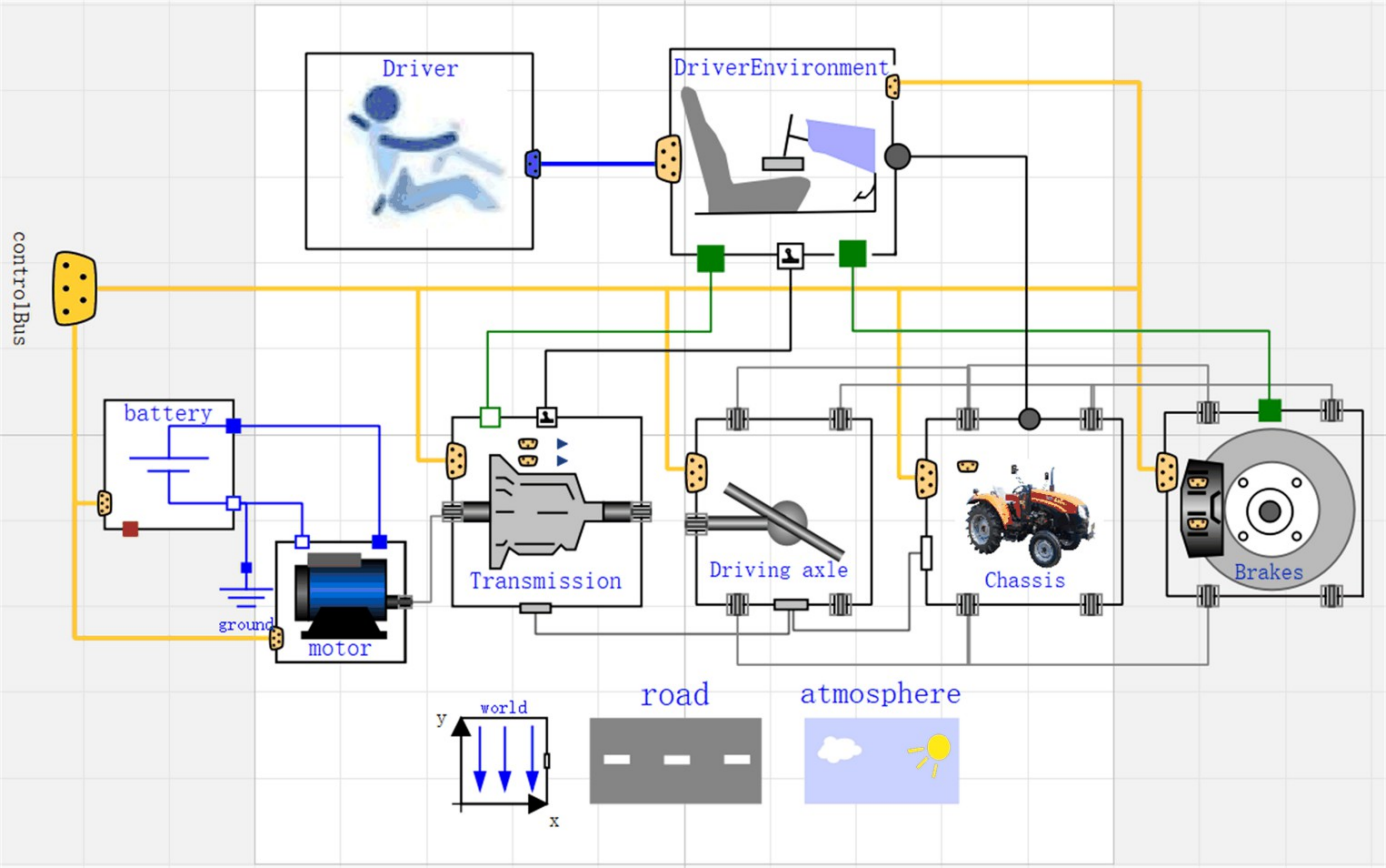
Simulation model of electric tractor drive system.

After completing the subsystem model of the electric tractor drive system, it also requires some accessory models such as road surface and environment to help build a complete simulation model of the electric tractor drive system. The accessory models involved in this study are obtained by porting and modifying the existing models in the VehicleInterface library. After the modules of the electric tractor drive system are established, according to the structural characteristics and coupling properties, the subsystem models are coupled and connected through the defined interfaces between the different subject areas. Fig 7 shows the simulation model of the electric tractor drive system.

In Fig 7, the blue lines represent the circuit part, which transmits electrical energy, the gray and green lines represent the mechanical part, which transfers mechanical energy, and the yellow lines represent the control part, which transmits control signals. world component is to provide global variables for the whole model, road component is to describe the basic information of the road, atmosphere component is to describe the environmental factors of the vehicle during operation to ensure the accuracy of the simulation results. The Driver component provides the braking demand, acceleration demand, gear mode, and power provision demand, and the DriverEnvironment component controls the clutch pedal, brake pedal, and the shifting process of the electric tractor.

### Electric drive system simulation model verification

Traction performance is the evaluation index of electric tractor performance. The transport condition is the evaluation index of the electric tractor operation ability. Therefore, according to the two indicators of electric tractor traction performance and transportation working conditions, validate the simulation model of the electric tractor drive system by combining simulation and experiment.

### Simulation verification

Set the environmental parameters of the simulation model as follows: operating slope is 0%, ambient temperature is 20 degrees Celsius, air density is 1.19kg·m^-3^, wind speed is 5km·h^-1^, and ground adhesion coefficient is 0.445. Since OpenModelica software uses a graphical interface, users can confirm their objects. According to the instructions, users set the relevant parameters based on the data in Table 1. The Driver module and the DriverEnvironment module set the simulation requirements to perform the maximum traction condition simulation.

**Fig 8:**
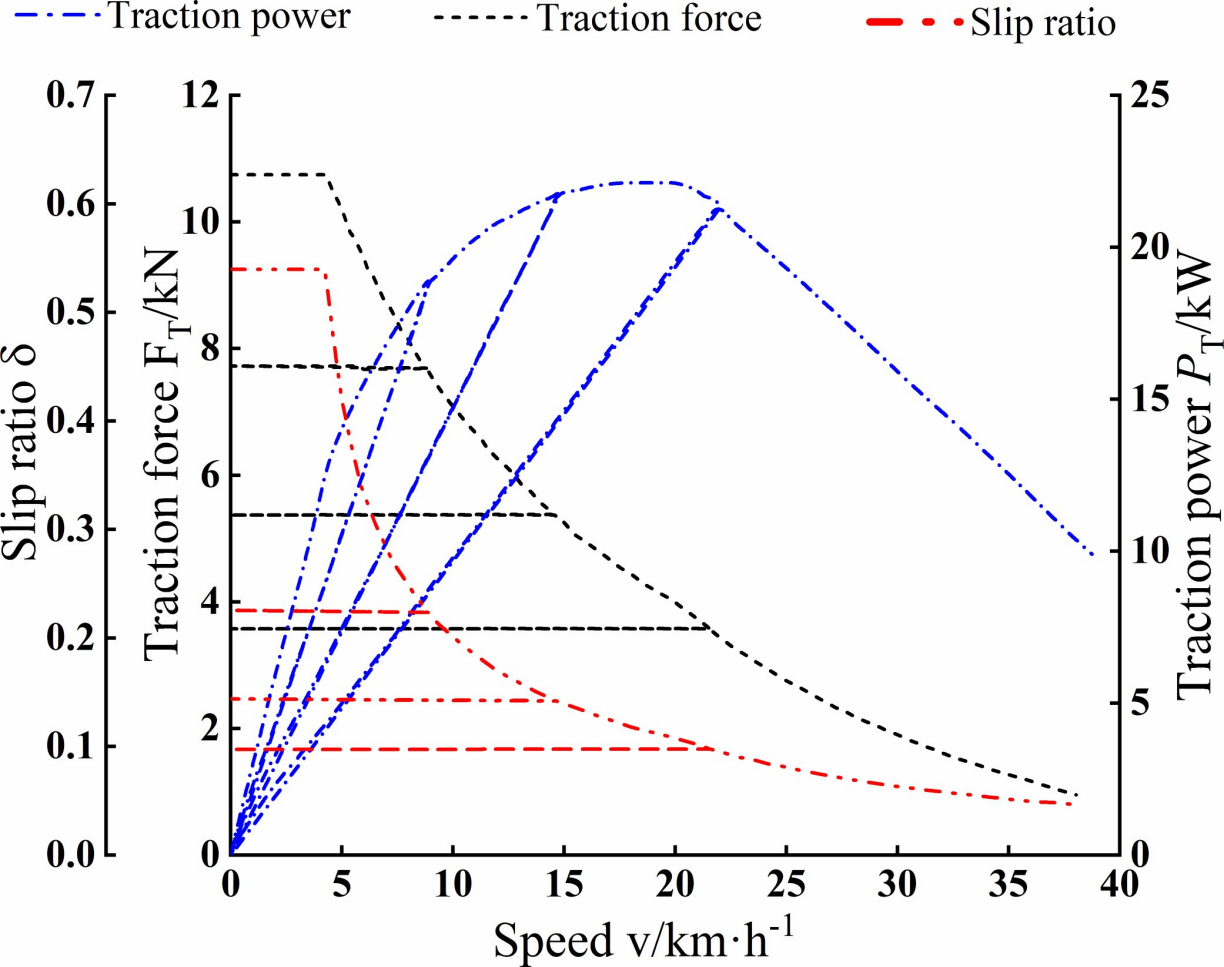
Simulation results of electric tractor traction performance.

Fig 8 is the simulation result of electric tractor traction performance, which is about the relationship curve of electric tractor speed with traction force, traction power, and slip rate. It is shown in Fig 8 that the maximum traction power in the working gear is 21.7kW, and the maximum traction force is 11kN. The maximum slip rate in the heavy-duty working gear is up to 0.55. The maximum slip rate in the transport gear is 0.1, which does not reach the characteristic slip rate.

**Fig 9:**
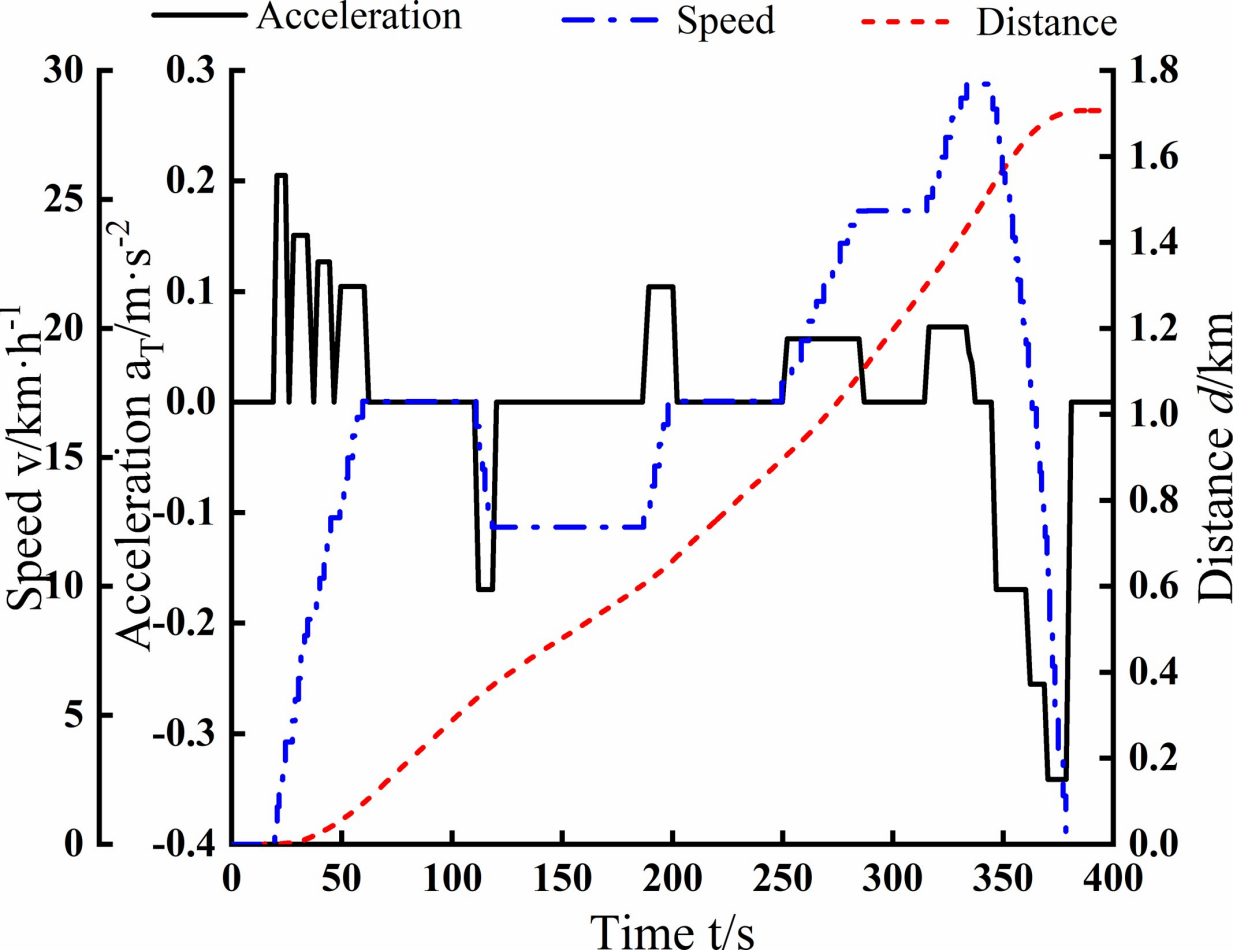
Simulation results of electric tractor transport conditions.

Fig 9 shows the simulation results of the electric tractor transport conditions. The simulation results are about the running time of the electric tractor with acceleration, speed, and distance curves. It is shown in Fig 9 that the running distance of the electric tractor is 1.72km and the speed range is 13~28km·h^-1^ in the simulation result of 400s transportation condition.

### Experimental verification

#### Traction test

**Fig 10:**
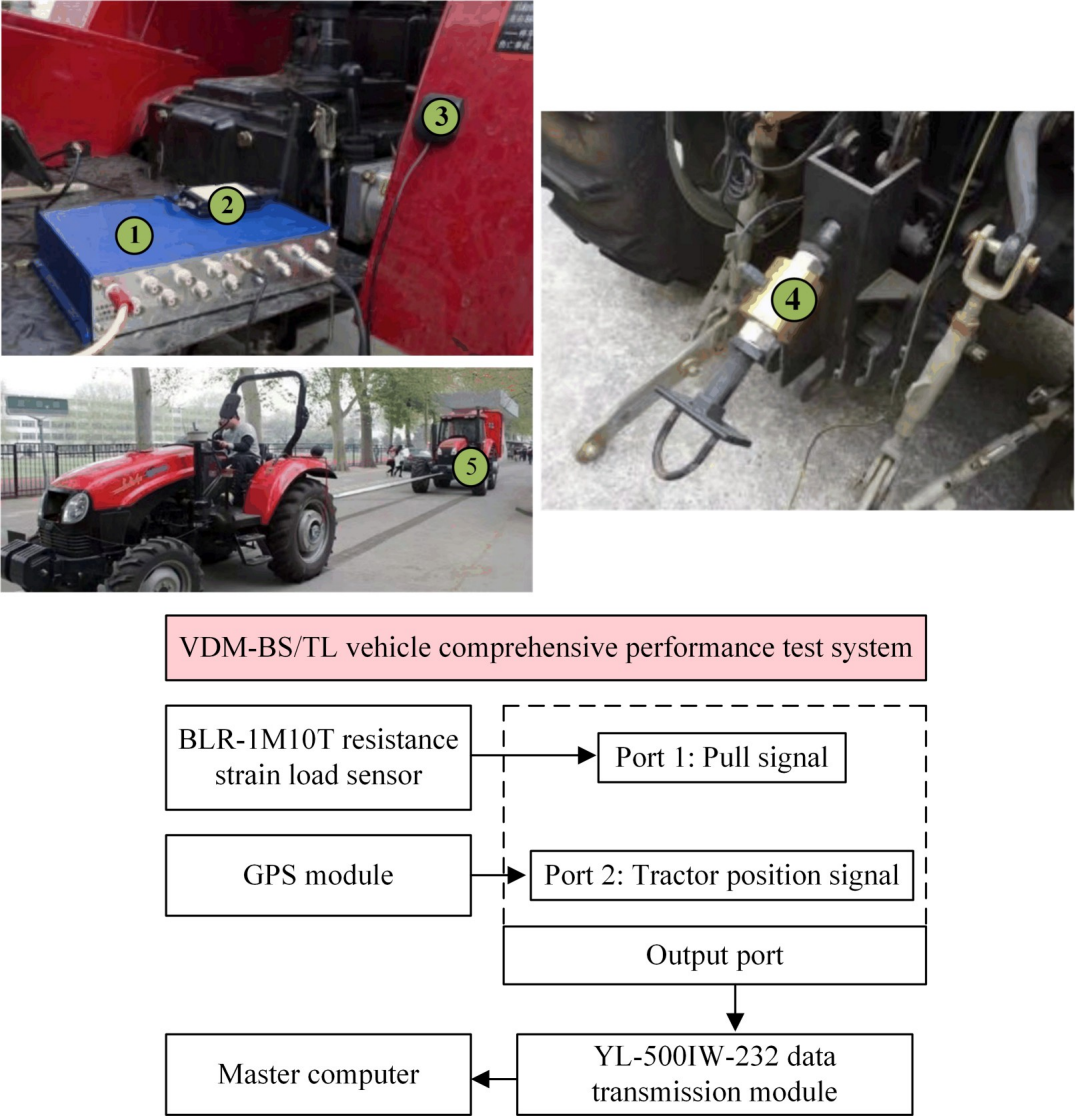
Traction test. (a) Data acquisition and positioning module, (b) Traction force comprehensive measurement module, (c) Traction performance test, (d) Block diagram of data acquisition, processing and transmission methods.

The effects of the overall vehicle parameters Wt and a on traction performance are not related to electrification. With the 100kW class load vehicle developed in the previous stage of the laboratory to test the traction performance under the whole machine parameters Wt and a. In Fig 10, ① is the vehicle integrated performance test system, ② is RF wireless digital transmission module, and ③ is the GPS module in Fig 10(A) data acquisition and positioning module. ④ in Fig 10(B) of the traction force comprehensive measurement module is the load sensor. ⑤ in Fig 10(C) of the traction performance test is the load car. Fig 10(D) shows the block diagram of data acquisition, processing, and transmission methods.

The test site was an outdoor horizontal concrete pavement. At the position of the tractor traction point, add the strain gauge pressure transducer to measure the traction point tension. By measuring the static angle between the traction point axially and the frame, calculating the horizontal force and normal force at the traction point according to the data measured by the strain gauge pressure transducer in the same step. During the test, the driver keeps the accelerator pedal open to 100%, and the load vehicle controls the traction force of the tractor under test through the eddy current retarder and sets the traction force increment by 100N in a single sampling step. Add the GPS module with frequency 1575.42MHz and rated voltage 3.0~5.0V to receive the position signal of the unit. In the vehicle comprehensive performance test system, processing the tension signal and unit position signal in a single sampling step derive the tractive force, slip rate, and vehicle speed values in a sampling step. The RF wireless digital transmission module uploads the measurement data to the upper computer side and calculates the traction power.

#### Test results

**Fig 11:**
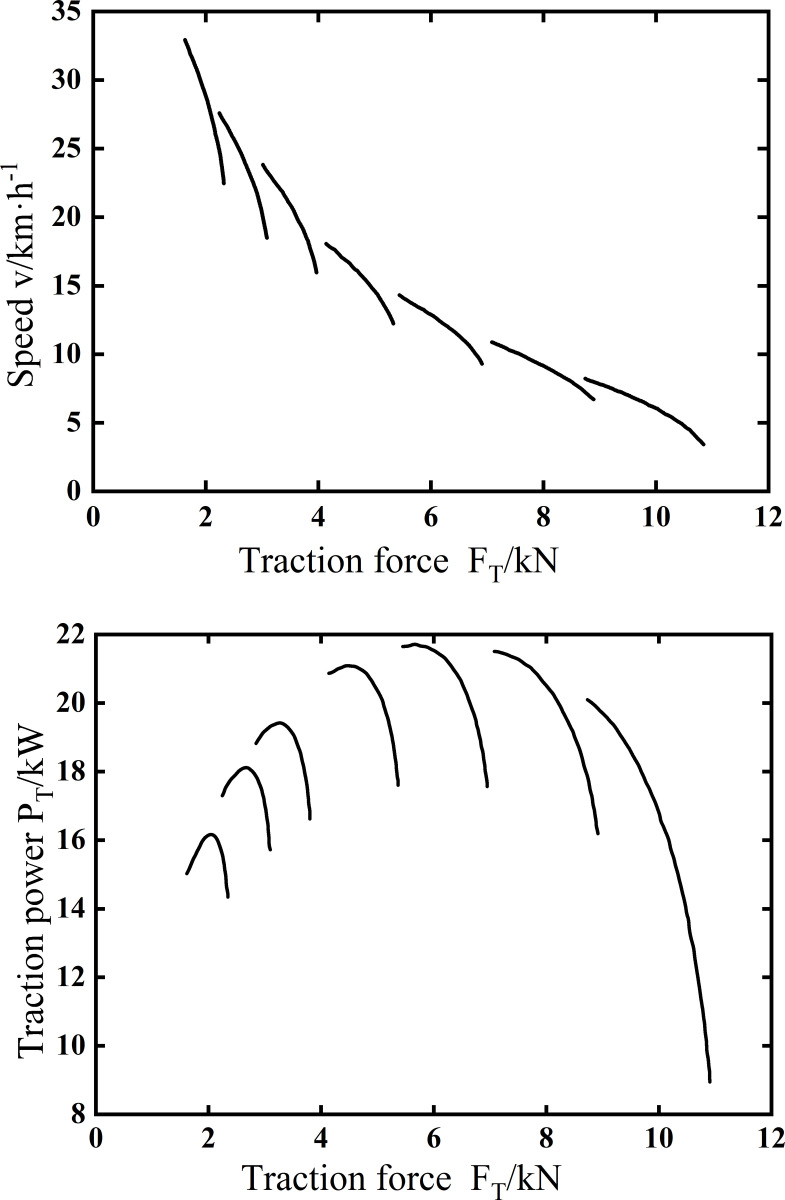
Traction test results. (a) Traction force-Speed, (b) Traction force-Traction power.

Fig 11 shows the traction test results of Traction force-Speed and Traction force-Traction power in working gear. It is shown in Fig 11 that the maximum speed is 9.2km·h^-1^, the maximum traction power is 21.7kW, and the maximum traction force is 11kN in working gear.

**Fig 12:**
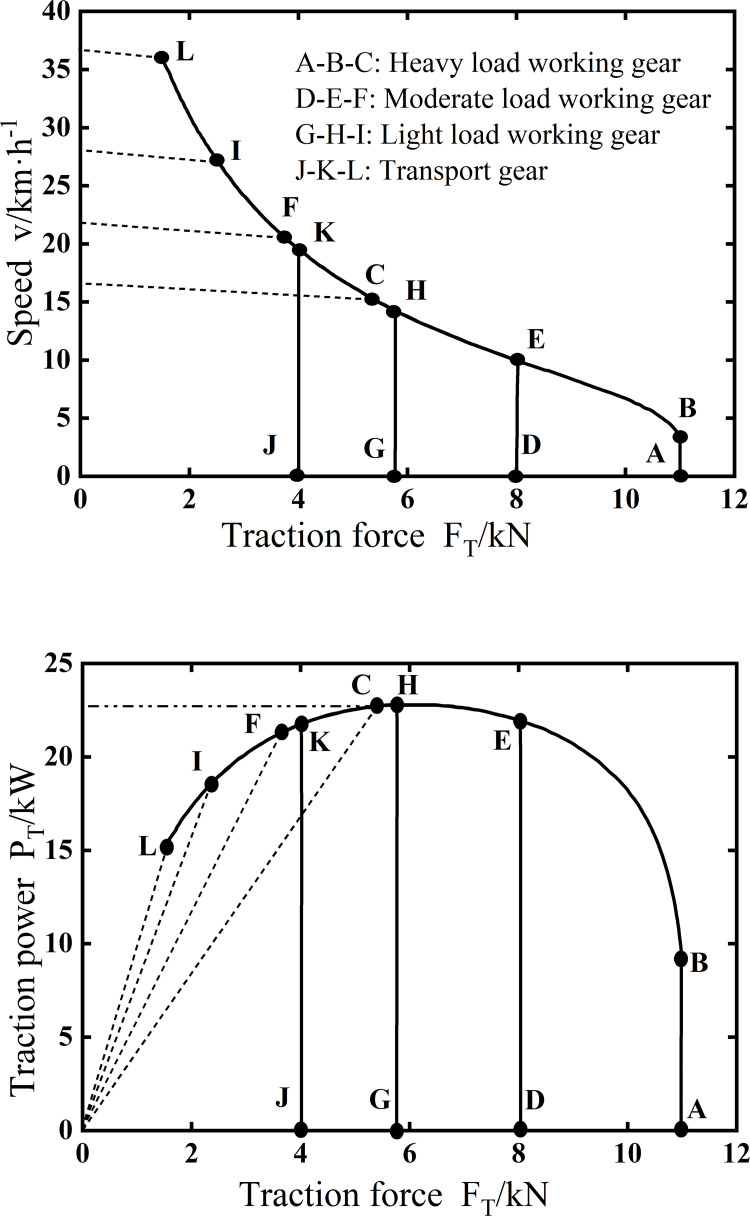
Traction performance. (a) Speed characteristics, (b) Traction power characteristics.

Fig 12 shows the traction performance of the electric tractor, where Fig 12(A) is the speed characteristics of traction force-speed, and Fig 12(B) is the traction power characteristics of traction force-traction power. As shown in Fig 12, the maximum speed of the electric tractor is 37km·h^-1^, the maximum traction power is 22.7kW, and the maximum traction force is 11kN. Compared with Fig 11, the electric tractor traction force-vehicle speed characteristic curve and traction force-traction power curve can cover the corresponding curve of the tractor, thus verifying the rationality of the drive system scheme designed in this paper.

After comparing Fig 8 with Fig 12(A), the curve of traction force and speed during operation in the corresponding gear decreases with the increasing speed of the vehicle, and the value of the maximum speed is also equal, 37km·h^-1^. After comparing Fig 8 with Fig 12(B), the maximum traction power values are equal in the working gear, 21.7kW. The maximum traction force values are also the same, 11kN. The simulation results for the transport conditions shown in Fig 9 are from 13 to 28km·h^-1^. Comparison with Fig 12(A) is consistent with the speed range of the electric tractor transport gear. In summary, through comparison between simulation and test, it is shown that the simulation results of the simulation model built based on Modelica are consistent with the test in terms of dynamic response and accuracy, thus verifying the credibility of the simulation and the correctness of the model.

## Conclusion

Modeling and simulation are critical to the research of electric tractors at this stage of their product development. Through modeling, simulation, and testing, this research explores and evaluates the electric tractor drive system. The following are the key conclusions:

According to the current development status of electric tractors, this study combines the application prospects of electric vehicles in the field of agricultural machinery, the overall scheme, and the parameters of the electric tractor drive system are designed and calculated based on the operational characteristics of the tractor.

According to the different subject areas of the electric tractor drive system, the subsystems are divided and established based on OpenModelica software, building a simulation model of the electric tractor drive system on this basis.

The performance of the electric tractor drive system is simulated and tested by two simulation tasks: traction performance and transportation conditions. The simulation results of the electric tractor drive system model built based on OpenModelica software are consistent with the test results, which verifies the credibility of the simulation and the correctness of the model built.

The methods and conclusions of this study have important guidance and reference significance for the subsequent development of new products of electric tractors. In the subsequent research process, the electric tractor hydraulic system can be established and integrated on the basis of this method to enrich and improve the model library of electric tractors for different professionals to test, analyze and evaluate the products.

## Supporting information

S1 Data (XLSX)
